# Broad protection with an inactivated vaccine against primary-isolated lethal enterovirus 71 infection in newborn mice

**DOI:** 10.1186/s12866-015-0474-9

**Published:** 2015-07-15

**Authors:** Junliang Chang, Jingliang Li, Xin Liu, Guanchen Liu, Jiaxin Yang, Wei Wei, Wenyan Zhang, Xiao-Fang Yu

**Affiliations:** Institute of Virology and AIDS Research, First Hospital of Jilin University, No 519, East Minzhu Avenue, Changchun, Jilin 130021 China; Department of Molecular Microbiology and Immunology, Johns Hopkins Bloomberg School of Public Health, 615 N. Wolfe Street, Baltimore, MD 21205 USA

**Keywords:** Enterovirus 71, Mouse model, Vaccine candidate

## Abstract

**Background:**

Circulating enterovirus 71 (EV-A71)-associated hand, foot, and mouth disease is on the rise in the Asian-Pacific region. Although animal models have been developed using mouse-adapted EV-A71 strains, mouse models using primary EV-A71 isolates are scarce. Lethal animal models with circulating EV-A71 infection would contribute to studies of pathogenesis as well as vaccine development and evaluation.

**Results:**

In this study, we established a lethal mouse model using primary EV-A71 isolates from patients infected with serotypes that are currently circulating in humans. We also characterized the dose-dependent virulence and pathologic changes of circulating EV-A71 in this mouse model. Most importantly, we have established this mouse model as a suitable system for EV-A71 vaccine evaluation. An inactivated EV-A71 vaccine candidate offered complete protection from death induced by various circulating EV-A71 viruses to neonatal mice that were born to immunized female mice. The sera of the immunized dams and their pups showed higher neutralization titers against multiple circulating EV-A71 viruses.

**Conclusions:**

Thus, our newly established animal model using primary EV-A71 isolates is helpful for future studies on viral pathogenesis and vaccine and drug development.

## Background

Human enterovirus 71 (EV-A71) is a non-enveloped, single-stranded positive-sense RNA virus that belongs to the *Enterovirus* species A genogroup in the *Picornaviridae* family. It began circulating in the Netherlands as early as 1963 and was first described in the USA in 1969 [1, 2]. EV-A71 and Coxsackievirus A16 (CV-A16) are the two major etiological agents that cause hand, foot, and mouth disease (HFMD); periodic large epidemics have occurred in recent decades, and it has become a severe public health problem [3–9].

Previous studies have shown that EV-A71 usually causes HFMD with severe neurological complications, including aseptic meningitis, brainstem encephalitis, poliomyelitis, encephalomyelitis, and even death [10–20]. In 1997, a large outbreak of HFMD caused by highly neurovirulent EV-A71 emerged in Malaysia and led to 41 deaths among young children [21]. In 1998, a large outbreak of enterovirus infection occurred in Taiwan that resulted in 405 severe cases in children and 78 deaths. Of the 78 children who died, 71 (91 %) were under 5 years of age [22]. In 2011, the largest recorded outbreak of EV-A71-associated HFMD occurred in mainland China, comprising >1.7 million cases and including 27,000 patients who exhibited severe neurological complications and 905 deaths [23].

EV-A71 has one serotype and can be classified into three genotypes (A, B, and C) and many subtypes (A, B0, B1-B5, and C1-C5). In Taiwan, the major subtypes of EV-A71 were C2 in 1998, B4 in the 2002 epidemic, C4 in the 2004-2005 epidemic, C5 in the 2006-2007 epidemic, B5 in the 2008-2009 epidemic, C4 in the 2010 epidemic, and B5 in the 2011-2012 epidemic [24, 25]. The predominant EV-A71 genotypes detected in Singapore were B3 in 1997-1999, B4 in 2000-2003, C1 in 2002, and B5 in 2006-2008. In mainland China in 1998-2011, all the strains were clustered in the C4 subgenotype of EV-A71.

Most research has been focused on developing vaccines against EV-A71 [26–35]. Given the successful experience in the development of inactivated whole viruses for poliovirus, influenza virus, and rabies virus, inactivated EV-A71 whole-virus vaccines have been produced by five manufacturers in mainland China, Taiwan, and Singapore. These vaccines have completed Phase III (mainland China) and Phase I (Taiwan and Singapore), respectively [32]. In mainland China, Beijing Vigoo Biological Co., Ltd (Vigoo), Sinovac Biotec Co., Ltd (Sinovac), and the Chinese Academy of Medical Science (CAMS) have used EV-A71 subgenotype C4 as a virus seed because it is the prevalent genotype in mainland China; however, Vigoo and Sinovac chose distinct strains, FY and H07, respectively, which were all isolated from Anhui province in South China [36, 37]. Thus far, no vaccine has effectively prevented EV-A71 infection in HFMD patients is available.

Previously, lethal mouse model in EV-A71 infection has been a pivotal evaluation role in the development of EV-A71 vaccines [27, 29, 33, 35]. However, EV-A71 viral isolates from HFMD patients in northeastern China [38] have not been previously studied in a mouse model or for vaccine development. Our group has isolated and identified several circulating EV-A71 strains from hospitalized HFMD children in northeastern China who had either severe or mild disease. We determined that these strains are complex recombinant viruses involving multiple type A human enterovirus (HEV) [38]. In the present study, we examined and compared the virulence, pathological changes, and progression induced by the circulating EV-A71 viruses, including Changchun (CC, Northeast China) and Fuyang (FY, South China) strains, in a neonatal mouse model. These strains showed different virulence, and a series of lethal strains could be used as a tool for vaccine evaluation. Furthermore, the EV-A71 vaccine candidate CC063 strain with the highest virulence also presented a broadly cross-neutralizing capacity and protection to neonatal mice from lethal-dose infect with various EV-A71 viruses. At the same time, the sera of the immunized dams and their pups showed higher neutralization titers against various EV-A71 viruses. The lethal challenge and protection in mouse model from circulating primary EV-A71 strains and the select vaccine candidates can be very helpful for vaccine development and evaluation.

## Methods

### Cells and viruses

African green monkey kidney epithelial cell line, Vero cells (Cat no. CCL-81; ATCC) were cultured in modified Eagle’s medium (MEM) (Gibco, Invitrogen, USA) containing 10 % fetal bovine serum (FBS) (Gibco, Invitrogen, USA) and 3 % L-glutamine at 37 °C with 5 % CO_2_. The CC strains of EV-A71 viruses were isolated from throat swabs of HFMD patients in Changchun, China in 2010. Viruses were continuously subcultured to the tenth passage in order to ensure stable titers and genetic features. FY0805 (GenBank accession No. HQ882182.1) and SHZH98 (GenBank accession No. AF302996.1) viruses were gifts from Dr. Qi Jin (Institute of Pathogen Biology, Chinese Academy of Medical Science). BrCr (GenBank accession No. U00871.1) viruses were received from Dr. PY. Mao (302 Military Hospital of China). CV-A16-CC024 is preserved in our laboratory and was isolated from a HFMD patient in Changchun, China (GenBank accession No. KF055238). Viruses were harvested when the cytopathogenic effect (CPE) reached 90 %, and the viral titers were determined in Vero cells by the microplate CPE method and calculated by the Reed–Muench method [39].

### Neonatal mouse infection model

One-day-old specific-pathogen-free (SPF) ICR neonatal mice (Experimental Animal Center, Jilin University) were used to establish the animal model of viral infection. All welfare and experimental procedures were carried out strictly in accordance with the guide for the care and use of laboratory animals and the related ethical regulations of the First Hospital of Jilin University. All efforts were made to minimize animal’s suffering.

The neonatal mice were divided into different groups, randomly and each group contains three litters (n = 8 ~ 10 per litter), and inoculated intracerebrally with EV-A71 viruses or MEM (10 μl/mouse), respectively. The survival rates and mean clinical symptoms were monitored daily for 21 days post-infection. The mean clinical symptoms were scored as follows: 0, healthy; 1, lethargy or weakness; 2, wasting; 3, limb shake; 4, paralysis in hind limb; 5, moribund or dead. The control mice were healthy throughout the experiments. The median lethal dose (LD_50_) was calculated as described by the Reed–Muench method [39].

### Histopathological and immunohistochemical analysis (IHC)

Three mice with grade 1 to 5 of clinical disease from each of three experimental groups, EV-A71CC063 (10^6.5^ CCID_50_/ml), BrCr (10^6.5^ CCID_50_/ml), EV-A71CC072 (10^6.5^ CCID_50_/ml), and three mice of grade 0 to 1 from the control group were subjected to histopathological and IHC analysis at 5 days post-infection. After the mice were anesthetized, tissues and organs, including lung, intestine, liver, kidney, hind limb muscle, spleen, heart, spinal muscle and brain, were harvested and immersion-fixed with 10 % formaldehyde solution for 5 days. Then, all the samples were dehydrated via an ethanol gradient, clarified through dimethylbenzene, and embedded in paraffin, and 4-μm sections were obtained for hematoxylin and eosin (HE) staining. For IHC examination, 4-μm sections of tissue samples were dewaxed and hydrated through an ethanol gradient. Antigens were then restored by microwaving for 15 min at 95 °C in citrate buffer. Used hydrogen peroxide (2.5 %) treatment to inhibit endogenous peroxidase activity of the samples. EV-A71 viral antigens were detected by anti-EV-A71 polyclonal antibody of rabbit (developed in our laboratory) and streptavidin-peroxidase anti-rabbit IgG kit (Fuzhou Maixin Biotechnology Development Co., Ltd, Fuzhou, China).

### Viral loads in newborn infected mouse tissues

Injected intracerebrally with EV-A71CC063 (10^6.5^CCID_50_/ml) or control medium, 12 viral challenged mice and 3 negative control mice were then used to detecting viral loads.

All samples including blood, lung, intestine, liver, kidney, hind limb muscle, spleen, heart, spinal muscle and brain were harvested from the experimental group (n = 3 per time point) on days 2, 4, and 6 post-infection and from the control group (n = 3) with no challenge on day 1. All the collected tissue samples were weighed individually and stored at -80 °C for further viral detection. The collected tissue samples were disrupted and homogenized in sterile phosphate buffer with the freeze-thaw/grinding method, then centrifuged. The clarified supernatants were collected, and viral loads in the tissue supernatant and blood were determined by real-time fluorescence quantitative reverse transcriptase PCR (qRT-PCR) and the results were expressed in log_10_ copies/ml of blood or log_10_ copies/mg of tissue. The 95 % confidence interval of the negative control values determined in various organs and tissues were regarded as the reference value for methodological sensitivity (0 copies/mg or ml).

### Inactivation, purification, and immunogenicity of EV-A71 virus candidate vaccine

The Vero cells infected by the seven strains of EV-A71 candidate virus were collected and centrifuged at 4500 × g for 30 min; the collected supernatants were inactived with formalin at 4 °C for 72 h. The effect of viral inactivation was tested by CPE, The inactived viruses were concentrated by ultrafiltration, following purified on Sepharose 4 Fast Flow gel. The protein concentration of the final virus elution was measured with a BCA kit (Thermo Scientific, Inc.), and the viral purity was determined by silver stain plus reagent (Bio-Rad, Inc.). The purified, inactivated EV-A71 derived viral proteins and negative control antigens prepared in similar fashion were mixed with alum and 0.5 ml (10 μg/ml) of the alum adjuvant vaccine was used for each immunization via intraperitoneal injection (I.p.).

Seven groups (n = 8 per group) of female adult ICR mice were immunized i.p. with a different EV-A71 vaccine candidate, CC063, CC072, CC077, CC080, CC085, SHZH98, or FY0805 two times at 2-week intervals. Serum was collected on the fourth week after immunization, and neutralization titers against the various EV-A71 viruses were measured by TCID_50_ assay in Vero cells.

### Serum neutralization test

Serum neutralization titers (NTAb) were determined by the TCID_50_ reduction assay in Vero cells. Isopycnic mixing (50 μl/well, respectively) of the serially diluted sera was carried out with the working concentration (100 TCID_50_/ml) variously circulating EV-A71 strain stocks; the mixtures were incubated at 37 °C for 2 h in 96-well plates. Subsequently, the Vero cells (2 × 10^5^/ml) were seeded onto 96-well plates (100 μl/well) for infection at 35 °C with 5 % CO_2_ and cultured for 7 days. The CPE of the Vero cells was observed by light microscopy. The highest serum dilution that could inhibit CPE in >50 % of the wells were determined as serum neutralization titers.

### Protective efficacy of vaccine-induced maternal antibody for neonatal mice

Eight-week-old female ICR mice (n = 3, each group) were injected i.p. with inactivated EV-A71CC063 vaccine or negative control phosphate-buffered saline (PBS) on weeks 0 and 2, respectively. The inactivated vaccine contained 10 μg of EV-A71CC063 virus. The first injection was 1 h after mate. After delivery, two experiments were carried out to confirm the protective efficacy of the humoral immune response of the EV-A71 vaccine candidate. First, three immunized maternal mice and their pups in the vaccine and negative control groups were euthanized, sera were collected for neutralizing antibodies assays against various EV-A71 strains. Second, four groups of pups (n = 3 litters per group, and n = 8 ~ 10 per litter) were intracerebrally challenged with different lethal doses of EV-A71 strains on day 1. Challenged mice were monitored for 21 days.

### Statistical analysis

The clinical scores, viral loads, and antibody titers were analyzed with nonparametric one-way ANOVA tests. The survival rates were determined by log-rank test. All statistical analysis were expressed as means ± the standard error of the mean (SEM). P < 0.05 was considered significant.

## Results

### Establishment of a neonatal mice model using primate EV-A71 viruses that produce strain-specific morbidity and mortality

**Fig. 1:**
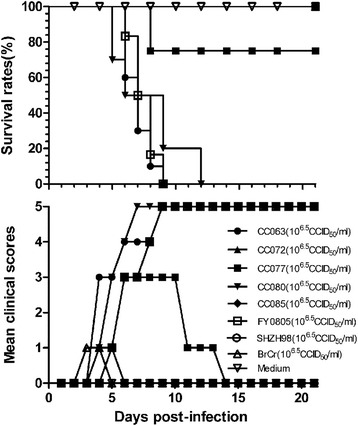
Distinct pathogenicity of circulating CC063, CC077, CC072, CC080, CC085, SHZH98, and FY0805 or BrCr viruses in neonatal mice. Newborn ICR mice were infected with indicated viruse (10^6.5^ CCID_50_/ml). Negative control mice were inoculated with MEM instead of virus. Survival rates and mean clinical scores were continually observed for 21 days after infection. The results are from three independent experiments, and each litter contained 8-10 mice. The mean clinical symptoms were scored as follows: 0, healthy; 1, lethargy or weakness; 2, wasting; 3, limb shake; 4, paralysis in hind limb; 5, moribund or dead

Although it has been determined that the dominant strains of EV-A71 have maintained a C4 genotype since 1998 in mainland China, there have been no reports of the development and use of a lethal animal model employing primary EV-A71 isolates. In 2010, multiple EV-A71 viruses (EV-A71CC063, EV-A71CC077, EV-A71CC072, EV-A71CC080, and EV-A71CC085) were isolated from infected HFMD patients in Northeast China by our group and determined to be recombinant forms of EV-A71 viruses [38]. To determine whether these EV-A71 viruses are lethal to neonatal mice and could be developed as a tool for vaccine evaluation, we intracerebrally injected multiple circulating EV-A71 viruses, i.e., CC063, CC077, CC072, CC080, CC085, FY0805, and SHZH98, as well as prototype EV-A71 BrCr (each at 10^6.5^ CCID_50_/ml), into one-day-old mice (Fig. 1). The clinical scores and survival rates of infected mice were monitored for 21 days. The results showed that the mice that were infected with CC063, CC077, CC080, and FY0805 became sick on day 4 post-infection and present a gradual aggravation tropism, with a clinical score of grade 5 on days 9, 9, 7, 9, respectively; and CC063, CC080, FY0805 infected mice all were dead by days 9, 12, 9, respectively, CC077 group had 78 % survival rate (Fig. 1). However, CC072, CC085, SHZH98, and BrCr did not cause mortality of the neonatal mice; the mice only showed some mild lethargy and inactivity in the early days of infection (mean, 2 ~ 7d) (Fig. 1). As expected, the mice of negative control group had a grade 0 of clinical score and 100 % survival rate.

### Lethal circulating EV-A71 viruses produce dose-dependent morbidity and mortality

**Fig. 2:**
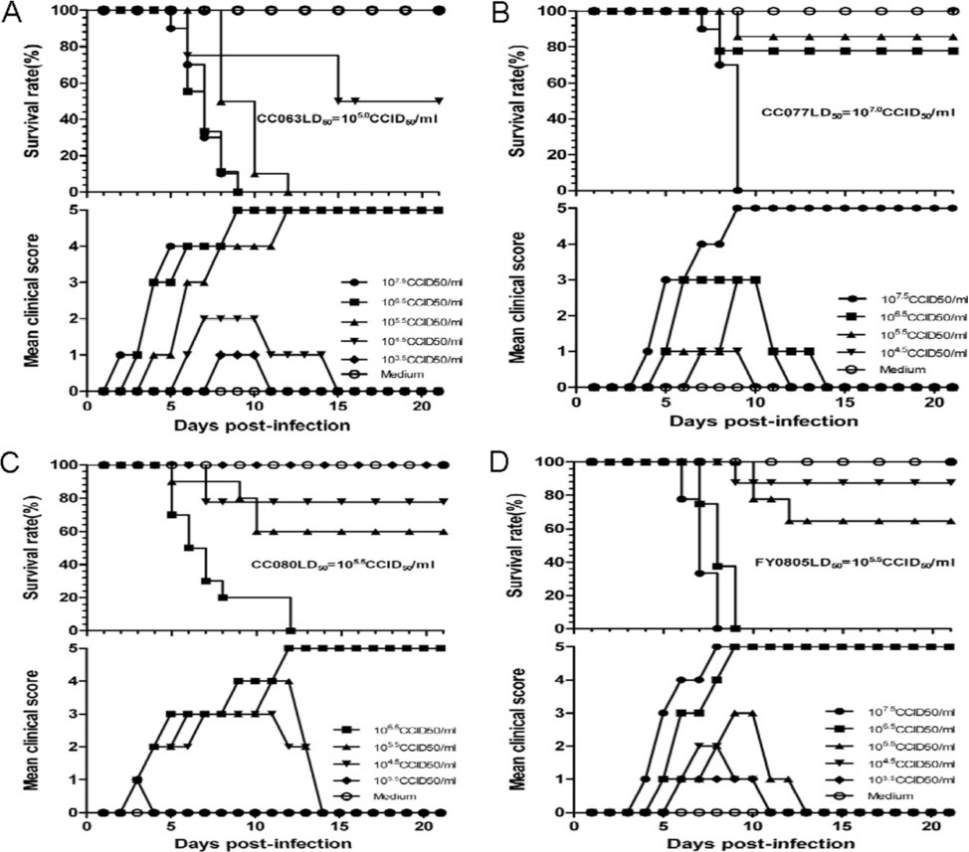
Circulating CC063, CC077, CC080, and FY0805 resulted in dose-related disease and mortality. One-day-old ICR mice (n = 8 ~ 10 per litter) were intracerebrally challenged with 10-fold serial dosages of viruses. **a** EV-A71CC063 challenged with 10^3.5^ CCID_50_/ml to 10^7.5^ CCID_50_/ml. **b** EV-A71CC077 challenged with 10^4.5^ CCID_50_/ml to 10^7.5^ CCID_50_/ml. **c** EV-A71CC080 challenged with 10^3.5^ CCID_50_/ml to 10^6.5^ CCID_50_/ml. **d** FY0805 challenged with 10^3.5^ CCID_50_/ml to 10^7.5^ CCID_50_/ml. Control animals were given medium instead of virus. Mortality and clinical symptoms were monitored and recorded daily for 21 days after infection. One representative from three independent tests is shown. The LD_50_ values are 10^5.0^ CCID_50_/ml, 10^7.0^ CCID_50_/ml, 10^5.5^ CCID_50_/ml, and 10^5.5^ CCID_50_/ml, respectively

Since the circulating EV-A71 viruses cause lethality in neonatal mice, we looked further into their virulence by measuring the LD_50_ of the viruses. Groups of one-day-old mice divided as described above were injected intracerebrally with 10-fold serial dilutions of CC063 (10^7.5^ ~ 10^3.5^ CCID_50_/ml), CC077 (10^7.5^ ~ 10^4.5^ CCID_50_/ml), CC080 (10^6.5^ ~ 10^3.5^ CCID_50_/ml), or FY0805 (10^7.5^ ~ 10^3.5^ CCID_50_/ml), with MEM as the negative control for each group (Fig. 2a-d). The mice that were infected with CC063 at titers of 10^7.5^ ~ 10^5.5^ CCID_50_/ml started to show symptoms on days 2, 3, and 4 post-infection, respectively; the symptoms were grade 1 and gradually became more severe until they reached grade 5 on days 9, 9, and 12, respectively (Fig. 2a). The three titers of CC063 (10^7.5^ ~ 10^5.5^ CCID_50_/ml) caused death beginning on days 5, 6, and 8, respectively, with a 100 % mortality on days 9, 9, and 12 post-infection, respectively (Fig. 2a). For CC063 at 10^4.5^ CCID_50_/ml, the virus caused a 45 % mortality in the infected mice, with a reversible mean clinical score of grade 2 (Fig. 2a). CC063 at 10^3.5^ CCID_50_/ml did not cause mortality, and it produced only a short period of grade 1 symptoms on days 8 ~ 10 (Fig. 2a).

For CC077, the challenged mice given 10^7.5^ CCID_50_/ml, 10^6.5^ CCID_50_/ml, or 10^5.5^ CCID_50_/ml began to sicken on days 4, 5, and 5, respectively, with a 0 %, 80 %, and 90 % survival rate (mean clinical score of grade 3), respectively (Fig. 2b). The challenged mice with 10^4.5^ CCID_50_/ml began to sicken on day 7 (grade 1), and no deaths occurred during the period of observation (Fig. 2b).

The mice that were infected with CC080 in various titers all became sick on day 3 post-infection (grade 1); after an increased tropism in clinical grade, only the 10^6.5^ CCID_50_/ml group reached grade 5 on day 12, whereas the 10^5.5^ and 10^4.5^ CCID_50_/ml groups had mean clinical scores of grade 3 during the whole test period (Fig. 2c). The challenged mice with 10^6.5^ ~ 10^4.5^ CCID_50_/ml began to die on days 5, 5, and 7, respectively (0 %, 60 %, and 80 % survival rate, respectively) (Fig. 2c). In the 10^3.5^ CCID_50_/ml group, no obvious symptoms or death of the infected mice were observed (Fig. 2c).

The mice challenged with 10^7.5^ CCID_50_/ml, 10^6.5^ CCID_50_/ml, 10^5.5^ CCID_50_/ml, or 10^4.5^ CCID_50_/ml FY0805 began to sicken on days 4, 5, 5, and 6 (grade 1), with 100 %, 100 %, 60 %, and 10 % mortality rates by days 8, 9, 12, and 9, respectively. The mice that were infected with the lowest dosage of 10^3.5^ CCID_50_/ml exhibited only mild symptoms (grade 1) on days 6 to 10 and no deaths (Fig. 2d).

In the aforementioned four experiments, no clinical symptoms or death were observed in the negative control group (Fig. 2a-d), indicating that the disease and death of the mice were specifically attributable to the EV-A71 viruses.

In conclusion, the symptoms and mortality rate produced by the four pathogenic virus strains increased in a dose-dependent manner. The median lethal doses (LD_50_) for CC063, CC077, CC080, and FY0805 were about 10^5.0^ CCID_50_/ml, 10^7.0^ CCID_50_/ml, 10^5.5^ CCID_50_/ml, and 10^5.5^ CCID_50_/ml, respectively. CC063 appeared to be the most pathogenic strain (Fig. 2). Thus, we have identified multiple circulating EV-A71 viruses that can induce strain-specific mortality and disease symptoms in newborn mice.

### Pathology in the mice post-infected with a lethal dose of circulating CC063

**Fig. 3:**
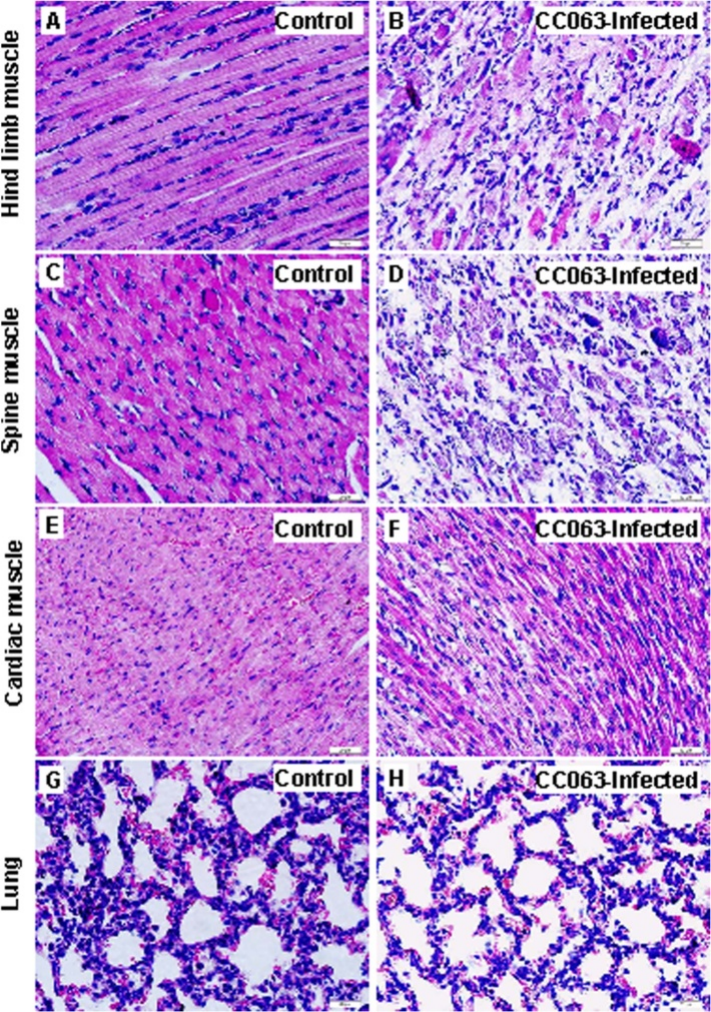
Pathological analysis of infected newborn mice after intracerebral challenge with a lethal dose (10^6.5^ CCID_50_/ml) of EV-A71CC063. One-day-old ICR mice were intracerebrally inoculated with EV-A71CC063 (10^6.5^ CCID_50_/ml) or medium (mock control). Representative images from the hind-limb muscle, spine muscle, cardiac muscle, and lung tissue at 6 days post-infection are shown. Infected mice (clinical grades 4-5) exhibited severe necrosis in the hind-limb muscle (**b**) and spinal skeletal muscle (**d**), but no obvious histological changes in cardiac muscle (**f**) or lung tissue (**h**) by HE staining. The results for non-infected mice were used as a control (**a**, **c**, **e**, and **g**). Magnification, 400 ×

The newborn mice challenged with any lethal dosage of EV-A71 virus all exhibited typical clinical symptoms such as wasting and hind-limb paralysis. To understand the pathological changes caused by CC strains in the neonatal mice, we selected the mice infected with the circulating CC063 strain, with strong virulence at the representative endpoint of grade 5 clinical score and conducted a series of pathologic analysis of multiple tissues to reveal changes that might be related to the death of the newborn mice. The results showed that the hind-limb muscle and spinal skeletal muscle fibers appeared severe necrosis, including muscle bundle fracture, muscle fiber swelling, and nuclear dissolution and shrinkage (Fig. 3b, d) when compared to those of the non-infected mice (Fig. 3a, c). However, no detectable pathological symptoms were found in the intestine, liver, spleen, kidney, or brain (data not shown). In addition, no obvious pathological changes were found in the heart or lungs (Fig. 3f, h). These results demonstrated that circulating EV-A71CC063 has a strong tropism cause severe lesions in the muscle tissues.

**Fig. 4:**
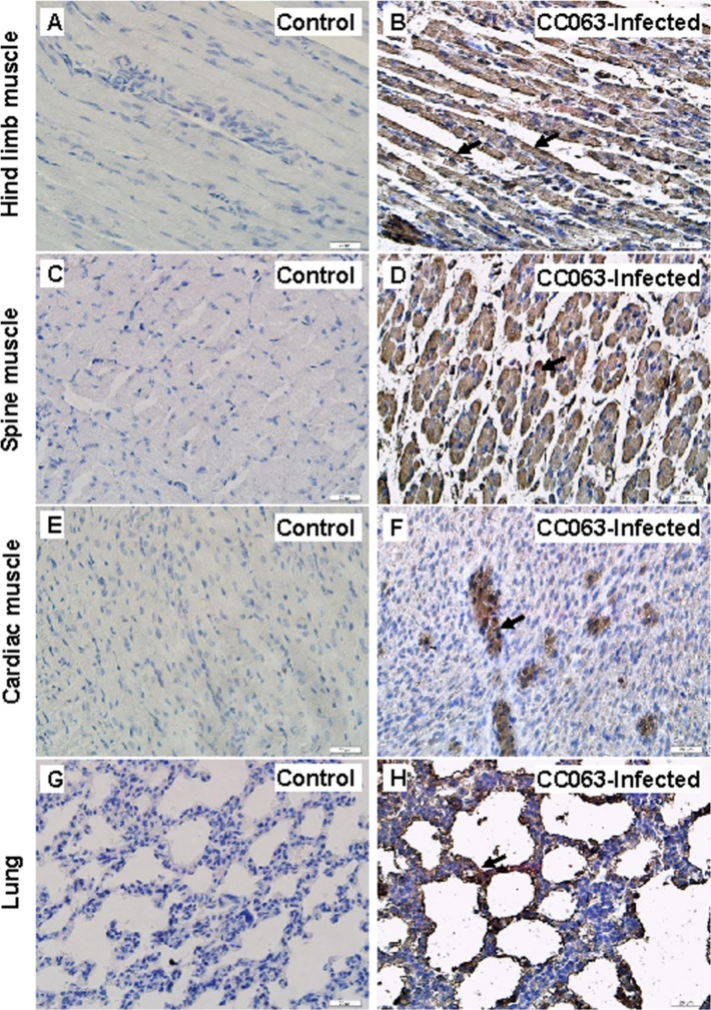
Immunohistochemical (IHC) staining results for infected mice after intracerebral challenge with a lethal dose (10^6.5^ CCID_50_/ml) of EV-A71CC063. One-day-old ICR mice were intracerebrally inoculated with EV-A71CC063 (10^6.5^ CCID_50_/ml) or medium (mock control). Representative sections from mouse tissues at 6 days post-infection (grades 4-5) are shown. The viral antigen (arrow) was detected in the hind limb muscle (**b**), spinal skeletal muscle (**d**), cardiac muscle (**f**), and lung tissue (**h**). In contrast, no viral antigens were detected in the corresponding tissues (**a**, **c**, **e**, and **g**) of the non-infected mice. Magnification, 400 ×

To better understand the distribution of viral antigen in selected tissues of CC063-infected newborn mice, we performed IHC staining of the associated tissues of the infected mice. The results showed widespread expression of viral antigen in the tissues, including the hind-limb muscle (Fig. 4b), spinal muscle (Fig. 4d), cardiac muscle (Fig. 4f), lung (Fig. 4h), and intestine, liver, brain, spleen, and kidney (data not shown). In contrast to the pathological changes in the hind-limb and spinal muscles, which expressed widespread viral antigen, we did not detect high viral antigen expression in the other tissues. No viral antigen was detected in the non-infected mice (Fig. 4a, c, e, g), confirmed that the detection was specific. Our results suggested that even CC063 could lead to widespread infection, but only induced pathogenesis in the hind-limb and spinal muscles of the mice.

### Viral loads in selected tissues of CC063-infected mice

**Fig. 5:**
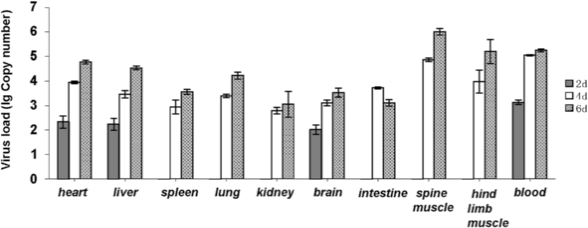
Kinetics of viral load levels in various tissues of EV-A71CC063-infected (10^6.5^ CCID_50_/ml) one-day-old ICR mice. The viral RNA copies were detected by qRT-PCR at days 2, 4, and 6 post-infection, respectively. The results represent the mean virus loads [(log_10_copies)/mg of tissue or/ml of blood] ± SD (3 mice per group, repeated 3 times). RNA samples from the mock-infected mice and positive virus culture medium were run simultaneously in each qRT-PCR, and known copies of the DNA fragments were used as standards to calculate the copy numbers of the viral RNA in the infected tissues

To further characterized the viral replication of CC063 viruses in infected mice, we determined viral loads in various organs at various time points post-infection (Fig. 5). Viral loads were first detected in the heart (10^2.32^ copies/mg), liver (10^2.23^ copies/mg), brain (10^2.01^ copies/mg), and blood (10^3.13^ copies/ml) at 2 days post-infection in the CC063-infected mice. Four days after challenge, the virus was detected in all tested tissues. The viral loads in various tissues of the infected mice, including heart, liver, spleen, lung, kidney, brain, intestine, spine muscle, hind-limb muscle, and blood reached higher levels of 10^3.94^ copies/mg, 10^3.46^ copies/mg, 10^2.94^ copies/mg, 10^3.39^ copies/mg, 10^2.79^ copies/mg, 10^3.11^ copies/mg, 10^3.73^ copies/mg, 10^4.87^ copies/mg, 10^3.98^ copies/mg, and 10^5.05^ copies/ml, respectively, on day 4 post-infection. This result suggested that the virus had spread systemically by day 4 post-infection. By day 6, the viral loads in all the tissues except the intestine had steadily increased, and the virus copy numbers were 10^4.78^ copies/mg, 10^4.54^ copies/mg, 10^3.56^ copies/mg, 10^4.23^ copies/mg, 10^3.05^ copies/mg, 10^3.53^ copies/mg, 10^3.10^ copies/mg, 10^6.01^ copies/mg, 10^5.20^ copies/mg, and 10^5.25^ copies/ml, respectively. In the negative control mice, no viral loads were detected in selected tissues, indicating that our results were specific. The highest viral loads were found in the spinal muscle, hind-limb muscle, and blood in the later stages (4 ~ 6 days). The detection of viral loads was also consistent with our IHC analysis (Fig. 4), suggesting that CC063 has a strong tropism for spinal muscle and hind-limb muscle.

### EV-A71CC063 vaccine candidate showed strongest immunogenicity and broader cross-neutralization capacity

**Fig. 6:**
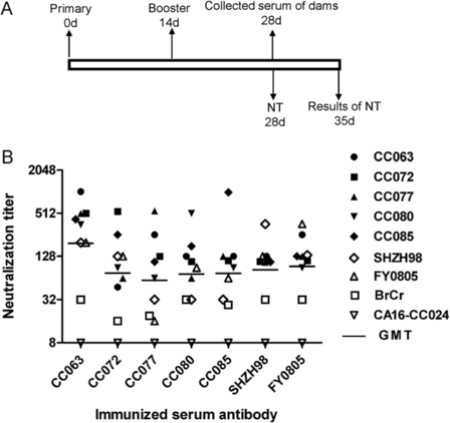
Serum cross-neutralization titers generated by seven EV-A71 vaccine candidates. Eight groups (n = 8 per group) of femal adult ICR mice were immunized with EV-A71 vaccine candidates including CC063, CC072, CC077, CC080, CC085, SHZH98, FY0805, and MEM (negative control) twice at 2 week intervals: **a** Diagram of the experimental procedure. **b** CC063 vaccine candidate produced highest mean cross-neutralization titers (GMT = 311). Mean cross-neutralization titers of the other EV-A71 strains were 100, 83, 111, 98, 113, and 117 (exclude CV-A16-CC024 and BrCr) for CC072, CC077, CC080, CC085, SHZH98 and FY0805, separately. The values for the negative control and CV-A16-CC024 sera were all < 8

According to the standards of innovative vaccine development reported by Liang et al [40], we compared an analyzed the immunogenicity of these EV-A71 strains including the CC strains, SHZH98, and FY0805. Eight groups of seven mice were immunized with various EV-A71 viruses with equal inactivated virus antigen (10 μg/ml and 0.5 ml/mouse) according to the immunization procedure as Fig. 6a. The antibody cross-neutralization titer (NT) was determined by using 28 days serum in cellular cytopathogenic effect method in vitro. As shown in Fig. 6b, CC063 vaccine candidate generated highest NT, and the geometrical mean (GMT) value was 311. The mean cross-neutralization titer value (exclude CV-A16-CC024 and BrCr) of CC072, CC077, CC080, CC085, SHZH98, and FY0805 were 100, 83, 111, 98, 113, and 117 (GMT). Moreover, serum of CC063 generated higher NT value against other virus strains except itself than other vaccine candidate (Fig. 6b). Through the comprehensive analysis of immunogenicity and broad neutralized feature, CC063 with the strongest virulence was chosen as an eligible vaccine candidate.

### Maternal antibody against the CC063 vaccine candidate can broadly protect the neonatal mice from lethal virus challenge

**Fig. 7:**
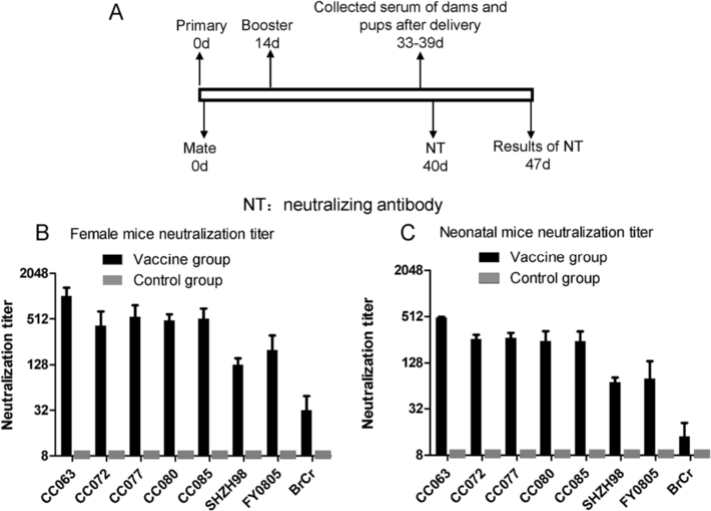
Broadly neutralizing antibody in immunized dams and pups. **a** Experimental design. (**b** and **c**) Adult female mice were immunized with inactivated EV-A71 viral preparation and subsequently examined for serum neutralizing antibody titers as described in section of the Methods. The sera were collected from 3 dams and their pups (8 to 10 mice per group, mixed) after delivery as described in section of the Methods

We hypothesized that the animal model developed in the present study may be a useful model for EV-A71 vaccine evaluation. To examine this hypothesis, we immunized female mice (n = 10) with the EV-A71 inactivated vaccine candidate and mated these mice after vaccine injection (Fig. 7a). Serum samples of three immunized dams and their pups on day 1 were collected. Neutralizing antibody (NTAb) titers against various EV-A71 strains were measured. The NTAb titers of female mice immunized against CC063, CC072, CC077, CC080, CC085, SHZH98, FY0805, or BrCr reached an average of 1024, 421, 550, 487, 518, 128, 199, and 32 (GMT), respectively. The NTAb titers of non-immunized mice were not detectable (Fig. 7b). The serum NTAb titers of pups born to the immunized female mice averaged 499, 263, 271, 247, 245, 71, 80, and 14 (GMT), respectively. As expected, no NTAb was detected in those pups born to non-immunized mice (Fig. 7c).

**Fig. 8:**
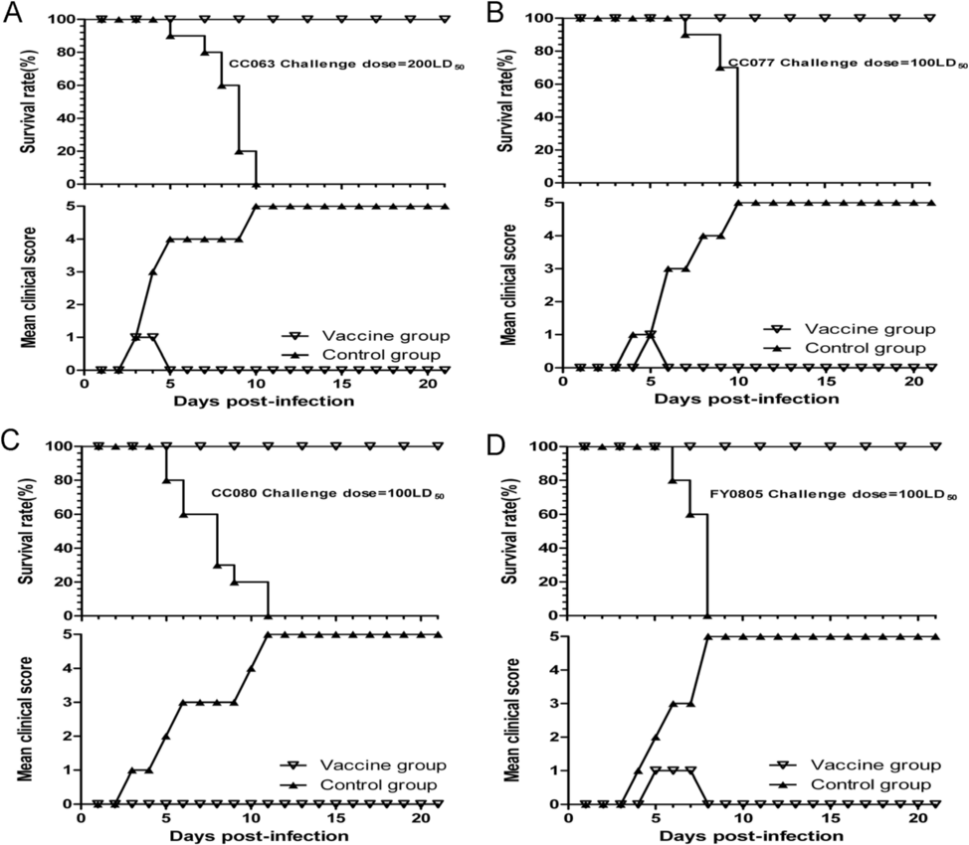
Broadly protection of maternal immunization with the inactivated EV-A71CC063 against lethal viruses challenge in neonatal mice. One-day-old ICR mice (n = 8 ~ 10 per litter) born to immunized mice were intracerebrally challenged with **a** CC063 (200LD_50_), **b** CC077 (100LD_50_), **c** CC080 (100LD_50_), or **d** FY0805 (100LD_50_) and had a 100 % survival rate during the test period of 21 days. The neonatal mice born to unimmunized mice (positive control) in the four groups all had a 100 % mortality rate and suffered increasing grades of clinical score

Next, we determined whether our EV-A71 vaccine candidate could protect neonatal mice from challenge with various lethal viruses. After delivery, we intracerebrally challenged the pups were with lethal a dose of CC063 (200LD_50_), CC077 (100LD_50_), CC080 (100LD_50_), or FY0805 (100LD_50_) on day 1. The neonatal mice born to unimmunized mice (MEM) in the four groups started to die an average of 6 days after virus inoculation, and all were dead by day 10 post-infection (Fig. 8a-d). In contrast, newborn mice of maternal immunized with vaccine candidate showed a 100 % survival rate and expressed lower clinical scores (grade 1) in the early stage of the tests (Fig. 8a-d). These results suggested that the animal model developed in this study can be used to evaluate EV-A71 vaccine candidates and that our inactivated EV-A71 vaccine candidate offers high efficiency and broad-spectrum cross-protection against diverse EV-A71 viruses in a neonatal mouse model.

## Discussion

Animal models plays a critical role in the development and evaluation of potential EV-A71 vaccines. Since normal adult mice are insensitive to EV-A71 infection, some researchers have suggested using neonatal mice or transgenic mice challenged with single viruses to evaluate the protective efficacy of vaccines [27, 29, 33, 35]. In our study, we have identified multiple lethal circulating EV-A71 viruses in a neonatal mouse model and have analyzed the pathogenic features of the lethal and non-lethal strains. These circulating EV-A71 strains were isolated from different regions of China. Early genome analysis demonstrated that these EV-A71 strains are circulating recombinant viruses [38] that are distinct from the prototype EV-A71 (BrCr). Interestingly, although the prototype EV-A71 (BrCr) did not cause lethal infection in the neonatal mice, several primary EV-A71 isolates readily generated pathogenic infections in these mice. On the other hand, some primary EV-A71 isolates could not cause lethal infection, despite the fact that they are genetically closely related to the pathogenic primary EV-A71 viruses.

Primary circulating EV-A71 viruses cause many symptoms in neonatal mice, including wasting, limb-shake weakness, and hind leg paralysis. HE staining showed severe lesions in the hind limb and spinal skeletal muscles, but not in the cardiac muscles or lung tissues; these findings are very different from those in the neonatal mice infected with lethal CV-A16 viruses, which suffered from severe lung damage [41]. We could also detected viral antigen in the hind limb, cardiac, and spinal skeletal muscles. As expected, a large amount of viral antigen was detected in the brain tissue (data not shown), a point that has been emphasized in many previous studies [11, 14, 17, 19, 42, 43]. Virus loads in these organs were consistent with our results. However, the expression of viral antigen could also be detected by IHC (data not shown) in many other tissues of the infected mice that showed tissue-specific pathogenesis.

Several viruses, such as CC072, CC085, SHZH98, and BrCr, were unable to induce neonatal death in the mice (Figs. 1 and 2). Consistent with the findings, pathological changes and viral loads could not be detected in the organs of these infected mice (data not shown), suggestnig that the absence of viral replication in the neonatal mice might be the main reason that the virus could not induce animal death. Our mouse model should be useful for future identification of viral determinants of pathogenic infection.

**Fig. 9:**
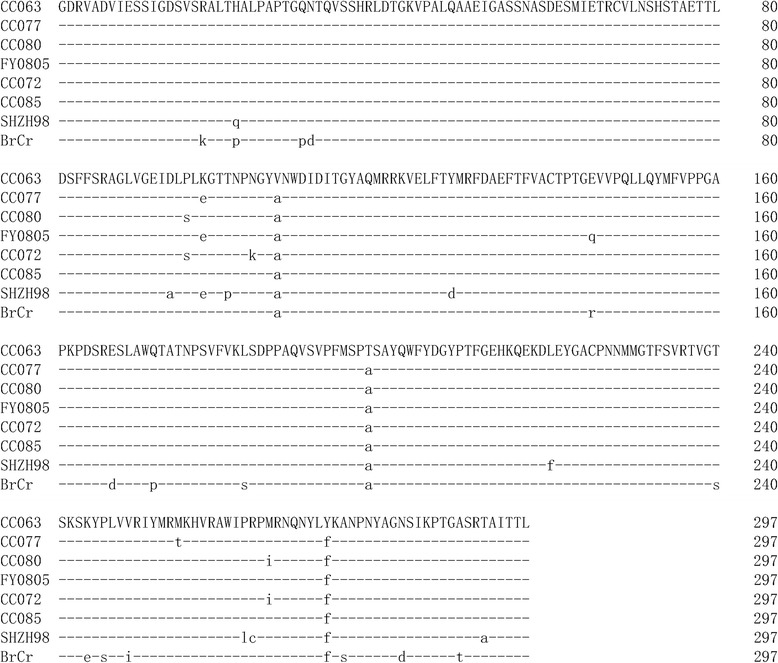
Amino acid sequences alignment of VP1 region of virulent (first four) and non-virulent (last four) EV-A71 strains

**Fig. 10:**
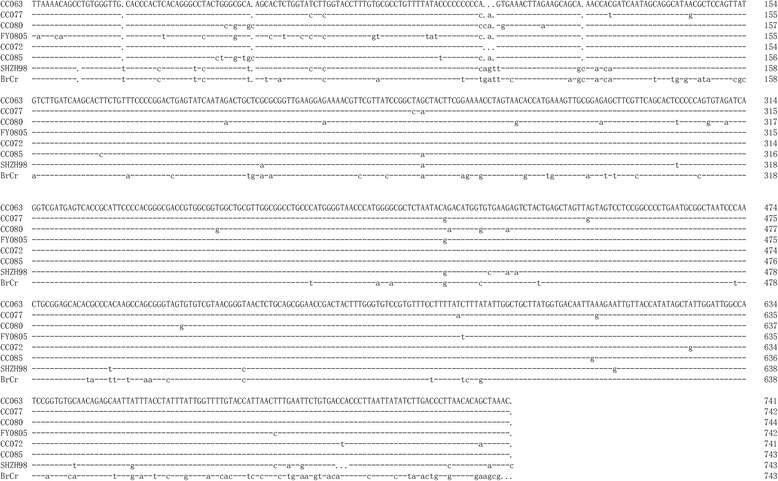
Nucleotide sequences alignment of 5’UTR region of virulent (first four) and non-virulent (last four) EV-A71 strains

Previous studies have demonstrated that 5’UTR and VP1 may be the major virulence associated regions of the EV-A71. We performed the 5’UTR and VP1 sequences alignment of the virulent and non-virulent EV-A71 viruses (Figs. 9 and 10). We found that the non-virulent EV-A71 contain mutations that are not detected in the virulent strains. Future studies will be required to determine whether these mutations influence virulence of EV-A71 in the mouse model. The eight EV-A71 strains evaluated in our mouse model have been associated with various disease symptoms in human. Only EV-A71 BrCr and CC063 have been associated with severe neurological diseases in people. Interestingly, BrCr is pathogenic in people but not virulent in the mouse model. It is possible that BrCr and CC063 could use different cellular receptors. The presence of certain cellular receptor in mouse allowed CC063 but not BrCr to replicate in the mouse model. Although the lethal mouse model may not be suitable for all EV-A71 strains, it is still useful for evaluating immune responses after EV-A71 vaccine immunization and protection of these immune responses against viral infection in this sensitive and convenient animal model.

Widespread HFMD has raised serious public health concerns in the Asia-Pacific region [4, 8, 9]. Coxsackievirus 16 (CV-A16) and enterovirus 71 (EV-A71) can both cause HFMD, major studies are focused on the latter as EV-A71 infection often associated with severe complications, including various neurological symptoms. Interestingly, we observed that administration of only the higher dosage of the various EV-A71 strains (CC063 LD_50_ ≈ 10^5.0^ CCID_50_/ml) could induce neonatal mouse death, as compared to circulating CV-A16 viruses (CC024 LD_50_ ≈ 10^1.65^ CCID_50_/ml) [41]. The pathogenesis induced by EV-A71 and CV-A16 needs to be further investigated in the future.

Our study demonstrates that primary isolates of circulating EV-A71 viruses differ greatly in their ability to elicit broad-range neutralizing antibodies. Using these viruses isolated from diverse areas, we characterized EV-A71 vaccine candidates with the highest virulence and determined that these candidates, derived from virus from Northeast China, could protect neonatal mice from challenge with diverse lethal strains. Moreover, we found that vaccine candidate CC063 had the highest virulence (LD_50_ ≈ 10^5.0^ CCID_50_/ml) (Fig. 2a), the strongest immunogenicity (Figs. 6 and 7), and the broadest cross-protection (Fig. 8). Therefore, using a sensitive mouse model and EV-A71 viruses, we have successfully studied the viral pathogenesis and identified a potential vaccine candidate.

## Conclusions

Since no effective antiviral agents are available, EV-A71 vaccine will play an important role in controlling EV-A71-induced HFMD in children. The selection of a vaccine candidate strain is the crucial factor, especially for the EV-A71 vaccine, because of its high mutation rate and frequent recombination [44]. In general, the virus vaccine candidate strain should have higher virulence, better immunogenicity, broad cross-protection, and genetic stability [37]. For vaccine evaluation, with the exception of the cellular CPE method in vitro, animal model systems can serve directly evaluation to protective efficacy and immunogenicity of candidate vaccines. Indeed, we observed high immunogenicity of the EV-A71 vaccine candidate both in vitro and in vivo. Even more important is the fact that the EV-A71 candidate vaccine in our study demonstrated broad cross-protection against lethal challenge with multiple EV-A71 viruses from the Jilin and Anhui provinces of China. Our study therefore provides important guidance for future HFMD vaccine development and evaluation which may lead to the discovery of potential vaccine candidates for the prevention and control of HFMD.
